# Supplementary material for: CD1d Selectively Down Regulates the Expression of the Oxidized Phospholipid-Specific E06 IgM Natural Antibody in Ldlr−/− Mice
Source: Antibodies (Basel). 2020 Jul 3;9(3):30. doi: 10.3390/antib9030030 (PMC7551411; doi:10.3390/antib9030030)
Supplement: Supplementary file 1 [file antibodies-09-00030-s001.pdf]

Supplemental Data

Material and Methods

Real time-PCR for E06 mRNA. RNA was isolated from splenocytes using the TRIZOL method and reverse transcribed using random primers and Superscript III (Invitrogen) according to the manufacturer's protocol. All reactions were performed in duplicate in a total reaction volume of 20 µl with qPCR Master Mix (Eurogentec, San Diego, CA) and 50 ng of cDNA, using the Rotor-Gene Q cycler (Qiagen). The primer sequences were designed based on PrimerQuest software (Integrated DNA Technologies, Coralville, IA) with modification for specificity. For the E06 IgH_V gene, the forward primer (5'-CTG TGC AAG AGA TTA CTA CGG TAG-3') flanked the E06 IgH V and D junction, the reverse primer (5'-AGG ACT GAC TCT CTG AGG AGA CG-3') flanked the JH and mu chain junction for amplifying E06 IgM, but not T15 IgA. The fluorescent probe (5'-CGC CCC AGA CAT CGA AGT ACC AG-3') matched to the E06 IgH D and J junction and was labeled with 5' reporter dye 6-FAM and 3' double-quencher ZEN/3'IBFQ (Integrated DNA Technologies, Coralville, IA). The expected amplicon size is 92 bp. The E06 expression levels were normalized to the reference gene GAPDH and analyzed by ΔΔCt and two standard curves relative quantitation methods using the Rotor-Gene Detection software (Qiagen).

Flow cytometry. Single cell suspensions of splenocytes or peritoneal cells in FACS buffer (1% BSA in PBS) were prepared and then stained with antibodies. Fluorochrome labeled antibodies against mouse CD3 (145-2C11), CD4 (GK1.5), B220 (RA3-6B2), CD25 (PC61.5), FoxP3 (FJK-16s), IL-4 (11B11), IL5 (TRFK5), IL-10 (JESt-16E3) and IFNγ (XMG1.2) were purchased from eBioscience. To assay for intracellular cytokine content, the isolated cells in complete RPMI 1640 medium were pre-incubated with 10 µg/ml LPS, 500 ng/ml ionomycin (LC

laboratories) and 50 ng/ml PMA (LC Laboratories) for 5 hours at 37°C. After washing, the cells were stained with cell-surface specific antibodies, followed by incubation with fixation/permeabilization buffer on ice for 30 minutes in the dark and then stained with specific intracellular cytokine-specific antibodies for 30 minutes on ice. Samples were analyzed on a FACSCanto flow cytometer (Becton Dickinson).

Figures and Figure Legends

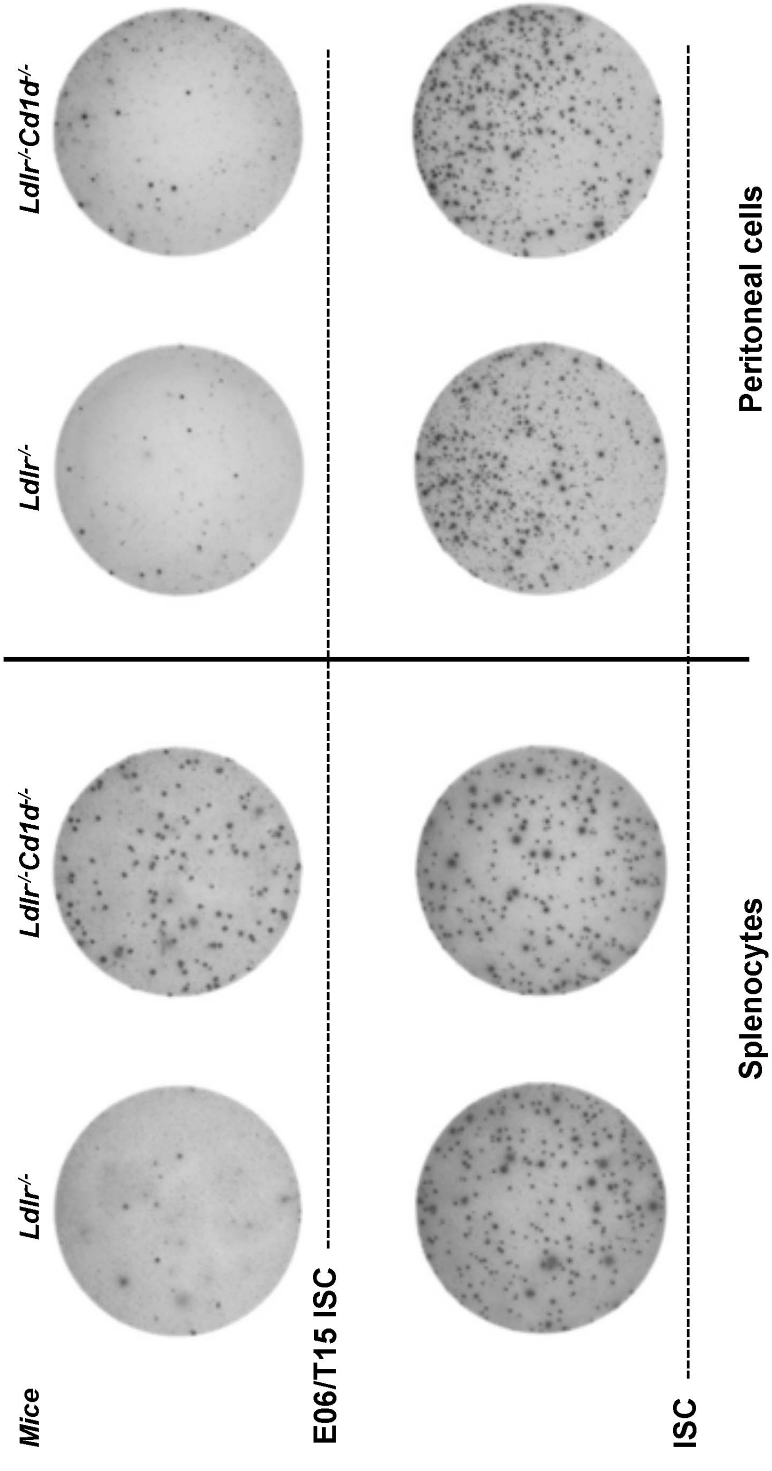
Mice
Ldlr-/-
Ldlr-/-Cd1d-/-
Ldlr-/-
Ldlr-/-Cd1d-/-
E06/T15 ISC
ISC
Splenocytes
Peritoneal cells

Figure S1. Representative ELISPOT assays. ELISPOT assays for E06 IgM secreting cells (ISC) and total ISC in the spleen and peritoneal cavity of chow-fed female *Ldlr*$^{-/-}$ and *Cd1d*$^{-/-}$*Ldlr*$^{-/-}$ mice are shown. E06 ISC are increased in the spleen of *Cd1d*$^{-/-}$*Ldlr*$^{-/-}$ mice.

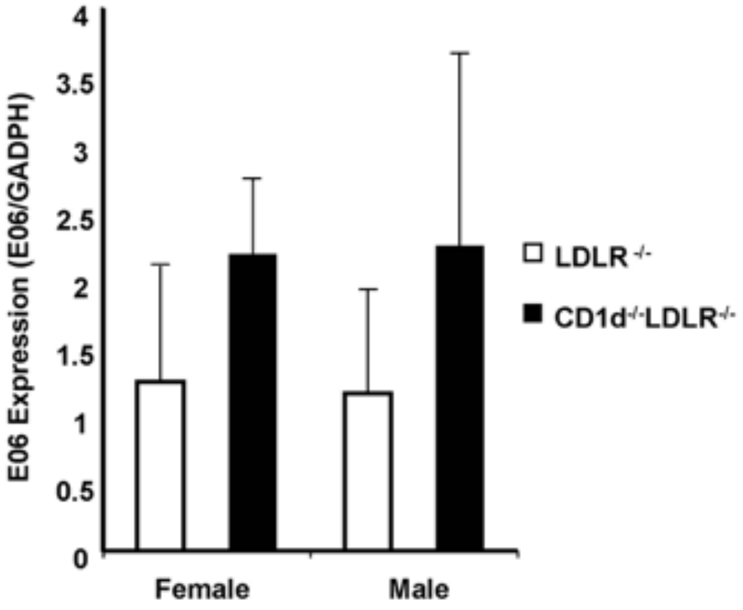

Figure S2. E06 mRNA levels in the spleen of female *Cd1d*$^{-/-}$*Ldlr*$^{-/-}$ and *Ldlr*$^{-/-}$ mice. Total RNA was isolated from splenic cells and the relative level of E06 mRNA normalized to GADPH was measured via real time PCR. The results are the mean ± SEM of n=3 *Ldlr*$^{-/-}$ and n= 4 for *Cd1d*$^{-/-}$*Ldlr*$^{-/-}$ mice. Data was analyzed by unpaired Student's t-test.

A.

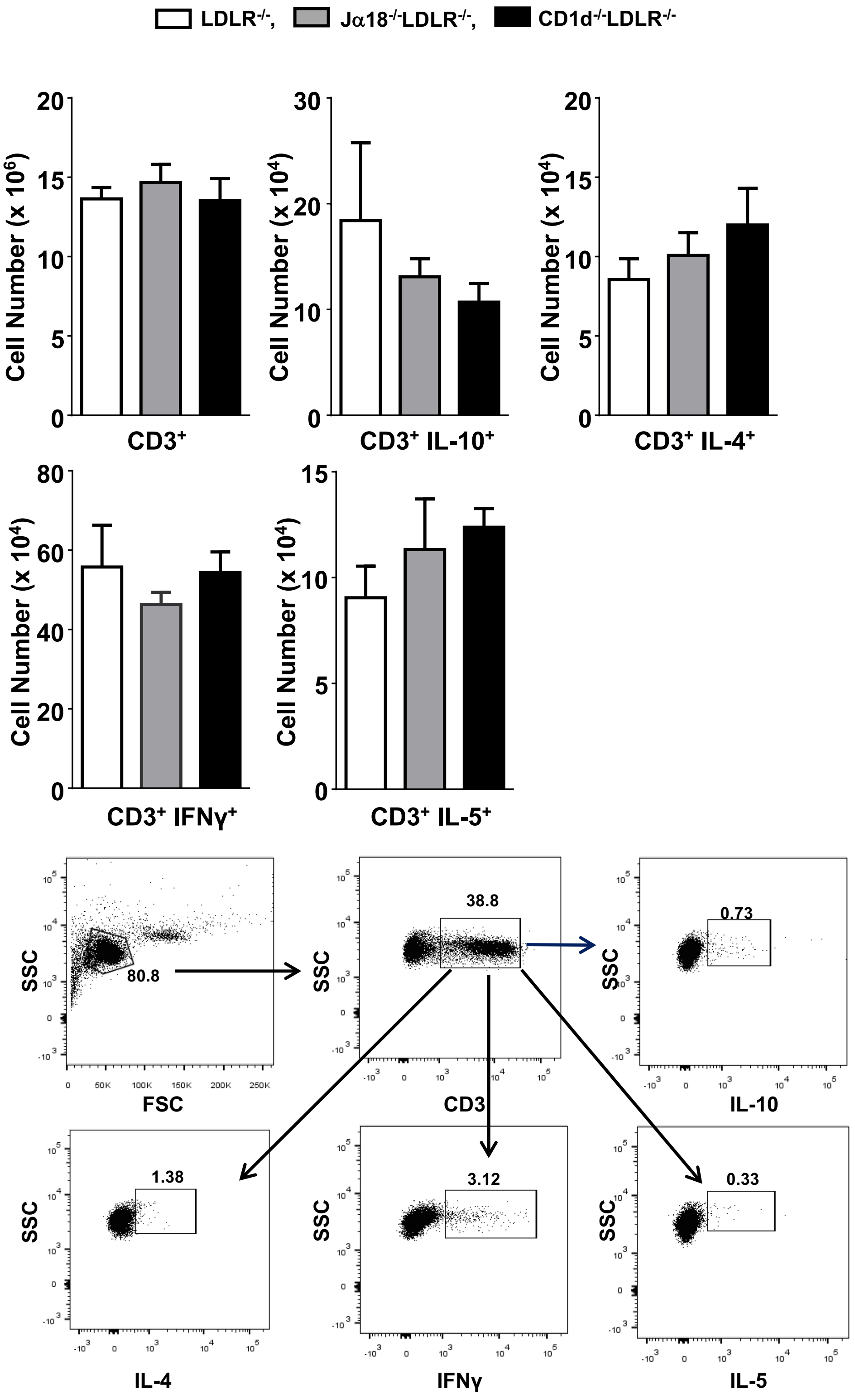
LDLR-/-, Jα18-/-LDLR-/-, CD1d-/-LDLR-/-
Cell Number (x 10^6)
CD3+
Cell Number (x 10^4)
CD3+ IL-10+
CD3+ IL-4+
CD3+ IFNγ+
CD3+ IL-5+
80.8
38.8
0.73
1.38
3.12
0.33
SSC
FSC
CD3
IL-10
IL-4
IFNγ
IL-5

B.

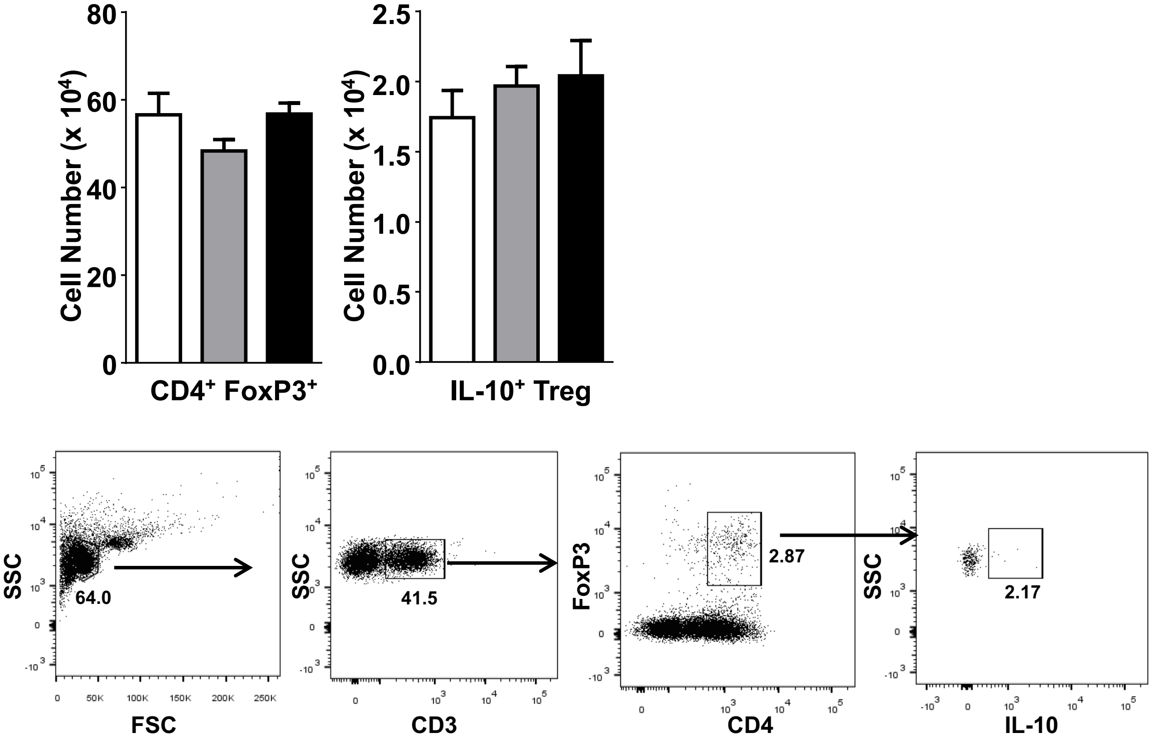

Figure S3. Neither T cells nor T_{reg} cells are decreased in the spleen of *Cd1d*$^{-/-}$*Ldlr*$^{-/-}$ mice. Splenocytes from female mice were analyzed for (A) total T cells ($CD3^+$ $B\text{-}220^-$) and cytokine producing T cells or (B) total or $IL\text{-}10^+$ positive T_{reg} cells ($CD4^+CD25^+$ $FoxP3^+$) cells by flow cytometry. The results are the mean ± SEM of n=9 pooled from two independent experiments in A and n=9-15 pooled from triplicate experiments in B. Data was analyzed by one way ANOVA followed by Bonferroni post-hoc test.
